# Expression of Markers Ki-67, Nestin, VEGF, CD34 and Apoptosis in Relatively Healthy Lung Tissue with Non-Changed and Metaplastic Bronchial Epithelium

**DOI:** 10.3390/medsci11010007

**Published:** 2022-12-29

**Authors:** Kaiva Zile Zarina, Mara Pilmane

**Affiliations:** Institute of Anatomy and Anthropology, Riga Stradins University, Kronvalda Boulevard 9, LV-1010 Riga, Latvia

**Keywords:** Ki-67, apoptosis, nestin, VEGF, CD34, bronchial epithelium

## Abstract

Background: Knowledge about the occurrence of processes such as proliferation, apoptosis and angiogenesis in healthy lung tissues with different bronchial epitheliums is limited, and further exploration can contribute to a better understanding of the physiological renewal of lung tissues. The processes mentioned above occur with the help of important tissue factors; therefore, the aim of the study was to determine the expression of markers Ki-67, nestin, CD34 and vascular endothelial growth factor (VEFG) and detect apoptotic cells in relatively healthy lung tissue. Methods: Samples of relatively healthy lung tissue were obtained from 19 patients and divided into groups of patients with non-changed and patients with metaplastic bronchial epithelium. Tissue samples were examined by hematoxylin and eosin staining. Ki-67, nestin, VEGF and CD34-positive cells were detected by the immunohistochemistry method. Terminal deoxynucleotidyl transferase (TdT) dUTP nick-end labeling (TUNEL) assay was carried out to detect apoptotic cells. The number of positive structures was counted semi-quantitatively by microscopy. Results: Ki-67-positive cells were detected in only one case. An occasional to moderate number of nestin-positive structures was found in various tissues of relatively healthy lungs with different bronchial epitheliums. No apoptotic cells were seen in non-changed bronchial epithelium, compared with few apoptotic cells in metaplastic bronchial epithelium. Metaplastic bronchial epithelium contained more VEGF-positive cells than non-changed bronchial epithelium. Samples with non-changed, and metaplastic bronchial epithelium both contained a similar number of CD34-positive structures. Conclusions: Proliferative activity and programmed cell death are not prominent events in normal lung tissue. A moderate number of nestin-positive cells in the alveolar epithelium and cartilage of bronchi with pseudostratified ciliated epithelium suggests a significant role of neuronal origin cells in these structures, to be intensified in metaplastic bronchial epithelium. A practically non-changed number of CD34-positive cells excludes any difference in stimulation of endothelial origin cells between lungs with different types of epithelium, while an increase in VEGF in structures with metaplastic epithelium suggests the presence/influence of tissue ischemia impact on possible development/maintenance of metaplasia.

## 1. Introduction

Proliferation, angiogenesis, vasculogenesis, cell death and other developmental processes in lungs are mostly described in association with certain pathological states, for example, malignity or tissue injury. Significantly less is known about the occurrence of events linked with renewal and development in healthy lung tissue, as well as about differences or similarities of distribution and frequency of such processes between lungs with pseudostratified ciliated epithelium and metaplastic—stratified squamous—airway epithelium. Gaining knowledge about the pattern of expression of proliferation and angiogenesis markers, neural and hematopoietic stem cell markers and frequency and distribution of apoptosis in healthy lung tissue can contribute to more efficient and thorough diagnosis of pathological conditions. Furthermore, understanding the mechanisms that encourage self-renewal and regeneration of lung cells can lead to new therapeutic perspectives regarding lung disease [1]. The processes mentioned above occur with the help of important tissue factors and can give apprehension about the origin of different epitheliums in lungs, especially in the case of bronchial epithelium metaplasia (change from pseudostratified ciliated epithelium to stratified squamous epithelium).

So, Ki-67 is a widely known nuclear protein encoded by the MKI67 gene. It is acknowledged as a cell proliferation marker since an increase in MKI67 expression is associated with cell growth and division [2]. Ki-67 is shown to be an independent prognostic factor for various tumors, such as breast cancer [3], soft tissue sarcoma [4] and lung cancer [5]. Ki-67 plays a role in heterochromatin organization [6] and detects nuclear activity in such a way.

Nestin is classified as a type IV intermediate filament (IF) protein and was initially characterized as a marker of neural stem cells [7]. Nestin plays an important role in survival and proper self-renewal of neural stem cells; however, it is demonstrated that this function of nestin does not require its structural incorporation into the cytoskeleton [8]. Nestin is also found in various tumors [9], although it is now known that nestin can be expressed in diverse tissues, such as bone marrow [10], testis [11] and skeletal muscle satellite cells [12]. To our best knowledge, no information can be found also about nestin-positive cells in lungs with different epithelial patterns.

Vascular endothelial growth factor A (VEGF-A) belongs to the VEGF protein family and is one of the five proteins that share structural and functional similarity, others being VEGF-B, VEGF-C, VEGF-D and placental growth factor (PlGF) [13]. VEGF-A is considered to be the main regulator of angiogenesis in such processes as embryogenesis and skeletal growth [14]. In lungs, VEGF-A is expressed mainly by alveolar epithelium [15]; however, macrophages and fibroblasts contribute to this process as well [16]. This factor distribution in the case of different bronchial epitheliums is not researched.

CD34 is a transmembrane glycoprotein and is most commonly referred to as a marker of hematopoietic stem cells (HSC) and hematopoietic progenitor cells [17]. It is also known that expression of CD34 is present on such cells as multipotent mesenchymal stromal cells [18], muscle satellite cells and epithelial progenitors [19]. Upon activation, CD34 relocates from the apical position to the junctions between endothelial cells [20], and this function could be important in the development of lung metaplasia.

Apoptosis is the process of programmed cell death that occurs under normal physiological conditions, such as embryogenesis, tissue homeostasis and immune system regulation, and under pathological conditions. In particular, as regards lung tissue, apoptosis has proven to be a key step in pathological processes such as fibrosis in idiopathic pulmonary fibrosis (IPF) [21] and cytokine-storm-mediated damage in acute respiratory distress syndrome (ARDS) [22]. Apoptosis can be induced by various physical and chemical stimuli [23], and assessment of apoptosis in lungs can give better insight into how tissue homeostasis is maintained between lungs with metaplastic and non-changed bronchial epithelium.

The aim of the study was to determine the expression and distribution of markers Ki-67, nestin, CD34 and VEFG and detect apoptotic cells in relatively healthy lung tissue with pseudostratified ciliated epithelium and stratified squamous epithelium.

## 2. Materials and Methods

### 2.1. Patients

Lung tissue material collected during post-mortem autopsy from nineteen patients aged 19 to 95 was examined. All samples were collected from a similar location in the lung—cartilage bronchi region. Samples were collected approximately 12 h after biological death had occurred. All patients included in the study were considered to be relatively healthy. Diagnoses that were seen most frequently were cardiovascular diseases and unintentional injuries caused by trauma such as cut wounds and brain and cranium injuries. The term “relatively healthy” was chosen since there might have been some medical episodes in patients’ life that could have had an impact on the development of metaplastic epithelium that the authors of this study have no information about. All tissue samples included in the study contained visible sections of intact airway epithelium. Patients were then categorized into two groups—patients with pseudostratified ciliated epithelium (14 patients) and patients with stratified squamous epithelium (5 patients). Exclusion criteria are the following: patients with medical history of respiratory conditions and failure and/or acute or chronic respiratory pathology, patients that have received medications that may have affected the results of the study, tissue samples with pathological findings other than metaplastic airway epithelium seen in routine histological analysis.

All experiments were examined according to the ethical standards laid down in the 1964 Declaration of Helsinki. This study was approved by the Ethical Committee of Pauls Stradins Clinical University Hospital dated 23 January 2013 (No. 230113-17L). The consent form of relatives was obtained in each case.

### 2.2. Microscopy

#### 2.2.1. Routine Microscopy

Soft tissue material pieces with an approximate size of 1 cm^3^ were taken during the autopsy and further fixed in a mixture of 2% formaldehyde and 0.2% picric acid in 0.1 M phosphate buffer (pH 7.2). After fixing, the tissue samples were rinsed in thyroid solution, containing 10% sucrose for 12 h. Following the rinsing, tissue specimens were embedded in paraffin. Six to seven micrometer (μm) thin tissue sections were cut to prepare them for staining. Routine histological staining with hematoxylin and eosin was used for each patient. All the tissue specimens were examined by bright-field microscopy. Metaplasia was detected with simple visualization. Samples that contained stratified squamous bronchial epithelium everywhere in the slide or in patches were classified as samples with metaplastic airway epithelium. Samples that had no stratified squamous epithelium and contained only pseudostratified ciliated epithelium were classified as samples with non-changed airway epithelium. No other criteria were used for the detection of metaplasia and patient division into the two groups.

#### 2.2.2. Immunohistochemistry

Nineteen (19) tissue sections in total were prepared for the detection of markers Ki-67, nestin, CD34 and VEGF by the biotin–streptavidin immunohistochemistry (IMH) method. The characteristics and parameters of the primary antibodies used in this study are the following: Ki-67 (Code: CMC27531040, rabbit, work dilution 1:100, Cell Marque, Darmstadt, Germany); nestin (Code: ab5968, rabbit, work dilution 1:250, Abcam, Cambridge, UK); VEGF (Code: ab1316, mouse, work dilution 1:200, Abcam); CD34 (Code: sc-19621, mouse, work dilution 1:100, Santa Cruz Biotechnology, Dallas, TX, USA); TUNEL (Code: 42134700, work dilution 1:10, Roche, Basel, Switzerland).

Negative and positive controls were provided to evaluate the specificity and sensitivity of the IHC. Positive control was carried out using already proved positive stainings for the specific marker: spinal ganglion of the pig for nestin [24], hyperplastic vocal noduli for Ki-67 [25], extirpated v. saphena magna for VEGF [26], subepithelial blood vessels of oral mucosa for CD34 [27]. Negative control was performed by excluding the primary antibody from the staining procedure.

Terminal deoxynucleotidyl transferase (TdT) dUTP nick-end labeling (TUNEL) assay is a method designed for detecting apoptotic cells that undergo extensive DNA degradation during the late stages of apoptosis [23].

#### 2.2.3. Immunohistochemistry Evaluation

The Leica DC 300F camera microscope was used for examination of the samples under bright-field microscopy. Conventional histological photographs were taken and then analyzed using the picture visualization program Image-Pro Plus.

A relative number of positive immunohistochemical structures was graded semi-quantitatively. Appearance and distribution of Ki-67, nestin and apoptosis-positive cells were examined in bronchial epithelium (both non-changed and metaplastic), alveolar epithelium and cartilage. Expression of VEGF was examined in bronchial epithelium (both non-changed and metaplastic), subepithelial blood vessels and alveolar blood vessels. Expression of CD34 was examined in subepithelial and alveolar blood vessels.

Immunoreactive (positive) structures seen in the visual field were then counted. The scale that was used for the semi-quantitative method contains the following values: 0—no positive structures (0%); 0/+—occasional positive structures (12.5%); +—few positive structures (25%); +/++—few to moderate number of positive structures (37.5%); ++—moderate number of positive structures (50%); ++/+++—moderate number of to numerous positive structures (62.5%); +++—numerous positive structures (75%); +++/++++—numerous to abundance of positive structures (87.5%); ++++—abundance of positive structures (100%) [25] (see Supplementary Materials, Figure S1).

### 2.3. Statistics

Statistical analysis was carried out using the statistical program SPSS Statistics 27 (IBM Company, Burbank, CA, USA). Acquired data were ranked as ordinal values, where 0 positive structures seen in the visual field were ranked with the value of 0, occasional positive structures (0/+) were ranked with the value of 0.5 and few positive structures (+) were ranked with the value 1.0. The highest possible number of structures—abundance of positive structures (++++)—was ranked with the value 4.0. Non-parametric statistical methods were used, and Spearman’s rank-order correlation was determined to evaluate the relationship between nestin-positive and apoptotic cells in samples with non-changed and metaplastic bronchial epithelium and the relationship between VEGF-positive and CD34-positive cells in samples with non-changed and metaplastic bronchial epithelium. Values of *p* < 0.05 were considered statistically significant.

Spearman’s rho (r_s_) value of 0.00–0.30 was considered a very weak correlation; 0.30–0.50, as weak; 0.50–0.70, as moderate; 0.70–0.90, as strong; and 0.90–1.00, as very strong. In the case of a strong positive correlation, the r_s_ value is closer to +1; in the case of a strong negative correlation, the r_s_ value is closer to −1 [28].

## 3. Results

### 3.1. Routine Histology

**Figure 1:**
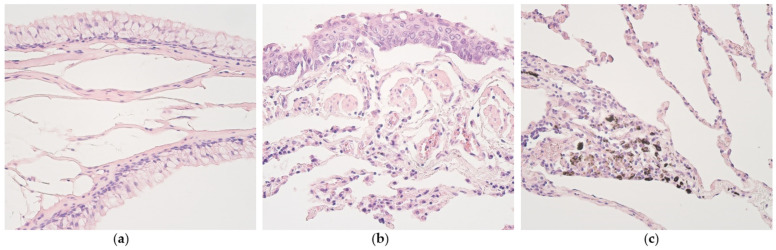
(**a**) Goblet cell hyperplasia seen in lung tissue of a 54-year-old male. Hematoxylin and eosin, X200. (**b**) Metaplastic bronchial epithelium seen in lung tissue of a 93-year-old male. Hematoxylin and eosin, X200. (**c**) Dust-containing macrophages seen in lung tissue of a 19-year-old male. Hematoxylin and eosin, X200.

Non-changed bronchial epithelium showed goblet cell hyperplasia in only one case (Figure 1a). Subepithelial edema was also found in one patient with metaplastic epithelium (Figure 1b). Emphysematous lung tissue regions were found in two patient materials. Dust-containing macrophages were seen in eight patients—six of them were patients with non-changed bronchial epithelium, and two of them patients with metaplastic bronchial epithelium (Figure 1c).

### 3.2. Immunohistochemistry

**Figure 2:**
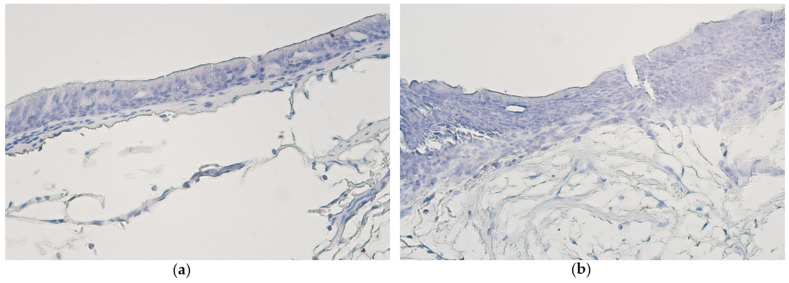
(**a**) No Ki-67-positive cells are found in non-changed bronchial epithelium of a 64-year-old male. Ki-67 IMH, X200. (**b**) All bronchial epithelium cells are Ki-67 negative in a metaplastic tissue material of a 61-year-old male. Ki-67 IMH, X200.

Ki-67-positive cells were detected in only one case—few Ki-67-positive cells were seen in bronchial epithelium of a patient with metaplastic airway epithelium. No positive structures were found in bronchial epithelium, alveolar epithelium or bronchial cartilage of all the other samples with either pseudostratified ciliated epithelium or stratified squamous epithelium (Figure 2a,b).

**Figure 3:**
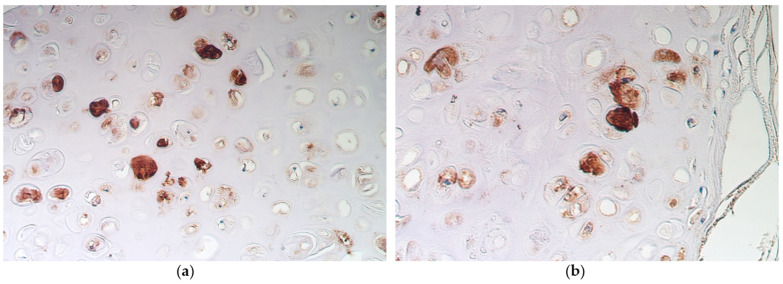
(**a**) Numerous nestin-positive structures in bronchial cartilage of a 54-year-old male with non-changed bronchial epithelium. Nestin IMH, X200. (**b**) Moderate number of nestin-positive structures in bronchial cartilage of an 86-year-old male with metaplastic bronchial epithelium. Nestin IMH, X200.

In non-changed bronchial epithelium, occasional nestin-positive structures were seen, compared with metaplastic bronchial epithelium, where a moderate number of nestin-positive structures was found (Table 1). In alveolar epithelium and bronchial cartilage, a moderate number of nestin-positive structures were detected in samples with non-changed bronchial epithelium (Figure 3a), as well as in samples with metaplastic bronchial epithelium (Figure 3b).

**Figure 4:**
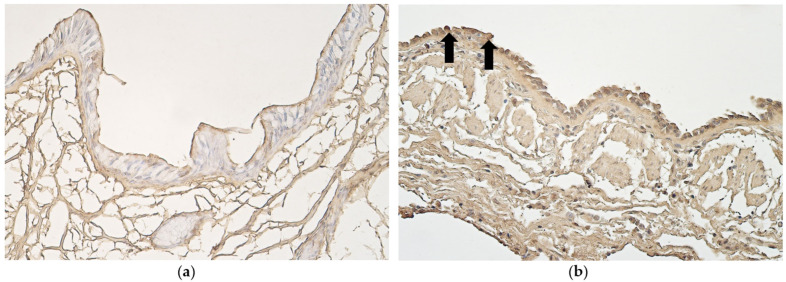
(**a**) No apoptotic cells in non-changed bronchial epithelium of a 54-year-old male. TUNEL, X200. (**b**) Few apoptotic cells in a 93-year-old male with partially detached metaplastic bronchial epithelium (arrows). TUNEL, X200.

No apoptotic cells were found in non-changed bronchial epithelium (Figure 4a). The same samples contained few apoptotic cells in bronchial cartilage. Few apoptotic cells could be seen in metaplastic bronchial epithelium (Figure 4b), while bronchial cartilage of samples with metaplastic epithelium contained occasional apoptotic cells. A moderate number of apoptotic cells was found in alveolar epithelium of samples with both metaplastic and non-changed epithelium (Table 1).

**Figure 5:**
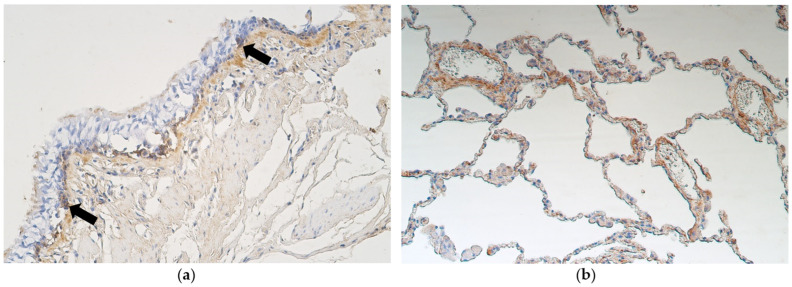
(**a**) Occasional VEGF-positive cells (arrows) are seen in non-changed bronchial epithelium of a 29-year-old male. VEGF IMH, X200. (**b**) Moderate number of VEGF-positive alveolar blood vessels and alveolar epithelial cells in 29-year-old male with metaplastic bronchial epithelium, VEGF IMH, X200.

Occasional VEGF-positive cells were seen in non-changed bronchial epithelium (Figure 5a). In the same samples, no VEGF-positive subepithelial blood vessels were found. In metaplastic bronchial epithelium, a moderate number of VEGF-positive structures was found, in the same samples few VEGF-positive subepithelial blood vessels were seen. Alveolar blood vessels of samples with non-changed bronchial epithelium contained a moderate number of VEGF cells, the same applies to the alveolar blood vessels of samples with metaplastic bronchial epithelium (Figure 5b) (Table 2).

**Figure 6:**
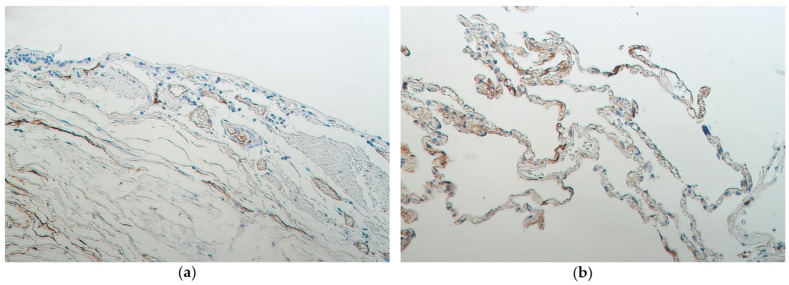
(**a**) Few to moderate number of CD34-positive subepithelial blood vessels in a 95-year-old female with partially detached non-changed bronchial epithelium. CD34 IMH, X200. (**b**) Few to moderate CD34-positive alveolar blood vessels in lung tissue of 86-year-old male with metaplastic bronchial epithelium. CD34 IMH, X200.

CD34-positive structures seen in subepithelial blood vessels of samples with non-changed bronchial epithelium varied (Figure 6a). In samples with metaplastic bronchial epithelium few CD34-positive subepithelial blood vessels were seen. Alveolar blood vessels of samples with non-changed and metaplastic bronchial epithelium both contained few CD34-positive cells (Figure 6b) (Table 2).

### 3.3. Statistical Data

Moderate positive correlation (r_s_ = 0.694) was seen between the number of nestin-positive and apoptotic structures among samples with non-changed bronchial epithelium. Moderate positive correlation (r_s_ = 0.598) was also seen between number of VEGF-positive and CD34-positive structures in samples with non-changed bronchial epithelium. Low positive correlation (r_s_ = 0.342) was seen between the number of nestin-positive and apoptotic cells in samples with metaplastic bronchial epithelium. Very weak correlation (r_s_ = 0.250) was found between the number of VEGF and CD34-positive structures among samples with metaplastic bronchial epithelium.

## 4. Discussion

In this study, we found no notable and systematic expression of Ki-67 in bronchial epithelium, bronchial cartilage and alveolar epithelium. This finding suggests that cellular proliferation is not prominent in relatively healthy lung tissue and/or also in metaplastic epithelium. It is known that an increase in bronchial epithelial proliferation can be seen within pathological conditions such as asthma [29] or chronic obstructive pulmonary disease (COPD) [30]. It is suggested that proliferation of airway epithelium may be triggered by a viral infection, injury or persistent airway inflammation. Exposure to allergens also contributes to increased cellular proliferation [31]. Research shows that epithelial Ki-67 index is related to smoking status—current smokers demonstrate higher Ki-67 levels than previous or never smokers; however, Ki-67 index decreases rapidly after quitting smoking; therefore, Ki-67 levels in former smokers were not greater than that in never smokers [32]. Thus, we hypothesized that Ki-67 expression would be higher in metaplastic bronchial epithelium in individual cases. Interestingly, squamous metaplasia can be defined as pre-neoplastic transformation of bronchial epithelium caused by toxic injury from cigarette smoke [30]. It is possible for squamous metaplasia lesions to regress back to normal epithelium and not undergo further transformation into neoplasia [33]. We may speculate that the absence of Ki-67-positive structures in most metaplastic bronchial epitheliums can be explained with association to the grade of metaplasia and probably the stage of metaplastic transformation. This was indirectly proved by Wang et al. [34] in 2006, who demonstrated various degrees of abnormal expression of Ki-67 in different types of bronchial epithelial dysplasia. It is worth noting that metaplasia is viewed more as an “adaptive” process in contrast to dysplasia, which is considered to be an “oncogenic” process [35]. During the process of squamous cell metaplasia, initial hyperproliferation of pseudostratified epithelium is seen when re-entering the cell cycle. Upon further differentiation, cells express markers of a squamous phenotype, for example, cytokeratins and a marker of terminal differentiation—involucrin [30]. It could be possible that epithelial cells reduce their proliferative activity after fully transforming into squamous stratified epithelium. However, to prove this hypothesis, an assessment of the stage of squamous metaplasia by detecting markers of a squamous phenotype should be performed. There might be various patterns of proliferation in different tissues, meaning that a specific appearance of Ki-67 may exist for some distinct type of epithelium. This proposal is supported by the absence of Ki-67-positive cells in normal oral lip tissue [36], minimal Ki-67 expression or complete absence in the normal cervical epithelium [37], as well as by research on Ki67 expression in columnar-lined esophagus, where different types of abnormal proliferation were described in metaplastic esophageal epithelium [38].

Non-changed bronchial epithelium contained almost no apoptotic structures, while in metaplastic bronchial epithelium, slightly more apoptotic cells were detectable. This suggests that cellular death simultaneously to proliferation is not prominent in relatively healthy bronchial epithelium; however, the dynamic and balance of both these processes can change during further squamous cell metaplasia, leaning towards more elevated levels of apoptosis. This study shows a more pronounced number of apoptotic cells in alveolar epithelium and only a small number of positive structures in cartilage of both non-changed and metaplastic bronchial epithelium samples. Apoptosis is a form of programmed cell death that occurs when specific cell membrane receptors—death receptors—are activated. Fas receptor (CD95) is a death receptor that is activated by Fas ligand (FasL) [39]. Research shows that the sensitivity of airway epithelial cells to FasL differs, where responsiveness to Fas-mediated apoptosis increases towards the distal regions of the lung. These findings explain that alveolar space could be more susceptible to apoptosis than the proximal airways such as bronchial epithelium [40]. Our study shows few to occasional apoptotic cells in bronchial cartilage. When compared with articular cartilage, the number of apoptotic structures was higher in the bronchial cartilage. Heraud et al. (2000) [41] reported that 2–5% of chondrocytes of normal articular cartilage (patients without osteoarthritis) showed apoptotic features. It appears that cartilage plasticity is not dependent on changes in soft tissues since no significant association was seen between chondrocyte apoptosis and the two different bronchial epitheliums.

We found a more pronounced number of nestin-positive structures in alveolar epithelium and bronchial cartilage of both specimens with stratified squamous epithelium as well as specimens with pseudostratified columnar epithelium. Although typically considered a marker of neural stem cells, nestin is now being viewed outside its original role. Nestin participates in signaling pathways that take part in differentiation, proliferation, apoptosis, motility, oxidative stress and migration [42]. Research suggests that nestin-positive cells have an impact on lung homeostasis. In our study, moderate positive correlation was found between nestin-positive and apoptotic cells in patients with non-changed bronchial epithelium. These findings suggest that nestin may take part in maintaining balanced lung function by affecting programmed cell death. A study performed by Ortega-Martínez et al. in 2014 [43] reports nestin-positive cells inside cartilage islets and cells undergoing division in close proximity to nestin-positive cells. Nestin-positive cells are also reported to be present in connective tissue associated with cartilage and in perivascular cells. Interestingly enough, the same study reports that chondrocytes were nestin-negative [44] Nestin-positive cells are detected in perivascular areas and connective tissues in close proximity to lung airway epithelium, as well as along the cells lining the epithelium. The authors of the study suggested that nestin-positive cells circulate in the bloodstream, transmigrate through blood vessels and localize in lung airway epithelium to take part in its turnover [45]. Thus, we also speculate that in our patients, nestin-positive cells are able to induce lung chondrocytes maintaining the nestin-positive phenotype. The role of nestin-positive cells in alveolar epithelium is similar to the role of such cells in bronchial cartilage and bronchial epithelium, and nestin-positive cells are a normal finding in most structures of relatively healthy lung tissue. More nestin-positive cells were detected in metaplastic bronchial epithelium than in non-changed bronchial epithelium. These results propose the idea that nestin participates in the metaplastic process possibly by regulating differentiation into stratified squamous epithelium. It is possible that pluripotent nestin-positive cells that can generate various types of lung tissue exist. This revelation can provide new therapeutic approaches for lung diseases [42]. Research shows that this marker is present in pathological conditions as well; for example, nestin contributes to progression of pulmonary fibrosis [46], and hypoxia-induced nestin promotes progression of non-small lung cancer cells by targeting specific transcription factors [47]. It was revealed in a study that nestin expression was robustly increased in the developing pulmonary vasculature, but nearly returned to levels observed under normoxic conditions at the late stage of pulmonary vascular remodeling in lung tissues of rats with induced pulmonary arterial hypertension (PAH). It was explained that nestin expression might be increased solely during proliferation. The change in nestin expression might represent a marker for pulmonary vascular remodeling [48]. A different study also points out that nestin overexpression directly promotes endothelial proliferation and angiogenesis in PAH, although it is not completely clear how nestin upregulation occurs in PAH [49]. Even though nestin can contribute to development of certain pathological states, it can still be considered a normal finding in healthy lung tissue. However, expression levels differ between physiological and pathological states.

This study showed a more distinct number of VEGF-positive structures in alveolar blood vessels of both specimens with non-changed and metaplastic bronchial epithelium and in subepithelial blood vessels of metaplastic bronchial epithelium and metaplastic bronchial epithelium itself. Previous studies confirmed that VEGF is abundant in lung alveolar epithelium, which is thought to be the main source of VEGF. Research suggests that VEGF is significant in maintenance of the balance of microvascular permeability in lungs—it impacts the capillary function and has permeability-enhancing effects. It is reported that VEGF acts as a paracrine and not as an autocrine factor since no apparent expression was previously seen in the endothelial cells [50]. Aside from its impact on endothelial cells, VEGF causes growth and differentiation in type II pneumocytes [51]. Medford and Millar (2006) [52] offer a hypothesis that VEGF has both protective and harmful activity in lungs—VEGF can protect and regenerate the epithelial surface in the case of a lung injury, yet it contributes to the generation of pulmonary edema if disruption of the alveolar–capillary membrane occurs as in acute respiratory distress syndrome (ARDS). The importance of VEGF in repair following lung injury can be explained by its ability to induce proliferation of the systemic vasculature and stimulate surfactant production. VEGF also has a part in regulating apoptotic cell clearance by alveolar macrophages; therefore, it affects the maintenance of tissue structure and function [53]. Since VEGF levels in normal lung tissue are noteworthy, however, angiogenesis is extremely restricted in the normal lung, and an explanation is sought. By the process of alternative RNA splicing, pro-angiogenic and anti-angiogenic isoforms of human VEGF-A are produced [54]. It is reported that anti-angiogenic isoforms of VEGF are expressed significantly by the alveolar epithelium and macrophages in a healthy lung; however, the expression was minimal in the ARDS tissue. Anti-angiogenic isoform VEGF_165_b has no proliferative effect on pulmonary endothelial cells; furthermore, it blocks the proliferative effect of pro-angiogenic VEGF165. These findings suggest that VEGF_xxx_b isoforms are a major part of VEGF molecules found in normal lung tissue, contrary to disease states such as ARDS [55]. It is known that VEGF is expressed by bronchial epithelial cells [56], although we noticed that expression levels were higher in metaplastic epithelium compared with non-changed bronchial epithelium. Factors that stimulate VEGF production are hypoxia and the presence of transforming growth factor β (TGF-β), interleukins and platelet-derived growth factors (PDGFs) [57], which probably could also be present in our patients with metaplastic bronchial epithelium. In 2012, Tanabe et al. [58] reported that TGF-β induces squamous metaplasia in airways. Presence of TGF-β might be the factor that connects squamous cell metaplasia and relatively increased VEGF production in the bronchial epithelium. VEGF levels in healthy lungs also increase as a response to ischemia. In metaplastic epithelium vascularization is modified—reduction of the amount of blood vessels in the squamous epithelium most probably impairs normal metabolism and promotes dysplastic alterations. This mechanism can explain the absence of blood vessels in the high-grade dysplastic epithelial layers [59]. It could be valuable to further explore how lung VEGF levels change simultaneously to multiple factors, for example, TGF- β and hypoxia, as well as how pro-angiogenic and anti-angiogenic VEGF isoforms are distributed among lung structures.

More consistent results regarding CD34 expression were seen in alveolar blood vessels of specimens with non-changed and metaplastic bronchial epithelium and subepithelial vessels of metaplastic bronchial epithelium, suggesting a more increased role of CD34 in the metaplastic change. In 2006, Pusztaszeri [60] reported that capillaries in alveolar wall stained for CD34. A different study revealed that capillary endothelial cells showed a stronger staining reaction than endothelial cells in arteries, veins, arterioles or venules, and staining intensity increased with age in veins and arteries and decreased with age in venules, arterioles and capillaries [61]. Research also suggests that vascular CD34 has a protective function during lung injury by enhancing endothelial–matrix interactions, thus maintaining the integrity of endothelial adhesion to the basal lamina and reducing permeability [62]. We found a moderate positive correlation between number of VEGF-positive and CD34-positive cells in specimens with non-changed bronchial epithelium; therefore, the two markers could be cooperating when regulating blood vessel permeability or taking part in new vessel formation.

All things considered, in lung tissue with non-changed bronchial epithelium, nestin was the most prominent marker found, and CD34 was also seen frequently. In lung tissue with metaplastic bronchial epithelium, the following markers were dominating: nestin, VEGF and CD34. These findings reveal that nestin and CD34 are stable markers when compared among non-changed and metaplastic epithelium. However, expression of VEGF differs between lung tissues with different types of epitheliums—this finding being the main distinction.

This study has several limitations, one of them being the relatively small number of patients. An equal number of non-changed bronchial epithelium tissue materials and materials with metaplastic bronchial epithelium would contribute to a more objective interpretation of mean values of tissue factors found and conclusions altogether. Results may differ between different age groups. An intriguing extension of this research would be describing tissue materials of smokers with different lung epitheliums. To obtain more objective results about tissue factor concentration polymerase chain reaction (PCR) tests or enzyme-linked immunoassay (ELISA) tests can be used, this technique could also give insight into gene transcription and translation ratio in different structures of lungs.

## 5. Conclusions

- Proliferative activity and programmed cell death are not prominent events in normal lung tissue with pseudostratified epithelium and in most cases of lungs with metaplastic epithelium. An exception is alveolar epithelium where apoptosis intensifies seemingly due to the specific aero-hematic barrier role.
- A moderate number of nestin-positive cells in the alveolar epithelium and cartilage of bronchi with pseudostratified ciliated epithelium suggests a significant role of neuronal origin cells in these structures, to be intensified in metaplastic bronchial epithelium.
- A practically non-changed number of CD34-positive cells excludes any difference in stimulation of endothelial origin cells between lungs with different types of epithelium, while an increase in VEGF in structures with metaplastic epithelium suggests the presence/influence of tissue ischemia impact on possible development/maintenance of metaplasia.

## Figures and Tables

**Table 1:** Appearance and relative number of nestin-containing and apoptotic cells in 19 relatively healthy lung tissue specimens.

	NESTIN			TUNEL		
	Bronchial Epithelium	Alveolar Epithelium	Cartilage	Bronchial Epithelium	Alveolar Epithelium	Cartilage
Patients with non-changed bronchial and alveolar epithelium						
Mean value	0/+	++	++	0	++	+
Patients with metaplastic bronchial epithelium						
Mean value	++	++	++	+	++	0/+

Abbreviations: “0”—no positive structures (0%) seen in the visual field; “0/+”—occasional positive structures (12.5%); “+”—few positive structures (25%) seen in the visual field; “++”—moderate number of positive structures (50%) seen in the visual field; “TUNEL”—TUNEL assay.

**Table 2:** Appearance and relative number of VEGF-containing and CD34-containing cells in 19 relatively healthy lung tissue specimens.

	VEGF			CD34	
	Bronchial Epithelium	Subepithelial Blood Vessels	Alveolar Blood Vessels	Subepithelial Blood Vessels	Alveolar Blood Vessels
Patients with non-changed bronchial and alveolar epithelium					
Mean value	0/+	0	++	Variable	+
Patients with metaplastic bronchial epithelium					
Mean value	++	+	++	+	+

Abbreviations: “0”—no positive structures (0%) seen in the visual field; “0/+”—occasional positive structures (12.5%); “+”—few positive structures (25%) seen in the visual field; “++”—moderate number of positive structures (50%) seen in the visual field; “VEGF”—vascular endothelial growth factor.

## Data Availability

The data presented in this study are available upon request from the corresponding author.

## Supplementary Materials

The following supporting information can be downloaded at: https://www.mdpi.com/article/10.3390/medsci11010007/s1, Figure S1: Visualization of quantifications used by photos of nestin-positive cells in bronchial cartilage
