# Supplementary material for: Expression of Markers Ki-67, Nestin, VEGF, CD34 and Apoptosis in Relatively Healthy Lung Tissue with Non-Changed and Metaplastic Bronchial Epithelium
Source: Med Sci (Basel). 2022 Dec 29;11(1):7. doi: 10.3390/medsci11010007 (PMC9844367; doi:10.3390/medsci11010007)
Supplement: Supplementary file 1 [file medsci-11-00007-s001.zip › medsci-2096239-supplementary.pdf]

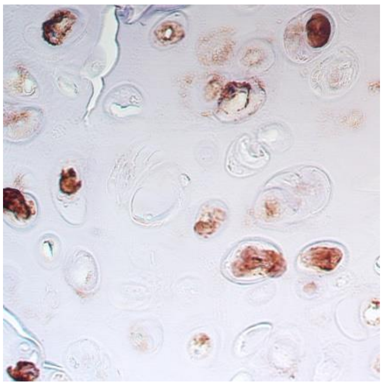

a

a) “++” - moderate number of positive structures (50%) seen in the visual field

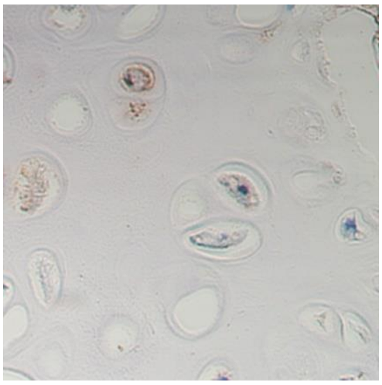

b

b) “+”- few positive structures (25%) seen in the visual field

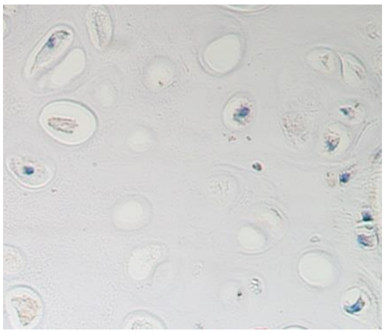

c

c) “0/+”- occasional positive structures (12,5%) seen in the visual field

Figure S1. Visualization of quantifications used by photos of nestin-positive cells in bronchial cartilage.
